# Antimicrobial resistance and phylogenetic lineages of KPC-2-producing blood-borne *Klebsiella pneumoniae* subsp. *pneumoniae* from Kolkata, India during 2015–2024: Emergence of *Klebsiella pneumoniae* subsp. *pneumoniae* with *bla*_KPC-2_, *bla*_NDM_, and *bla*_OXA-48-like_ triple carbapenemases

**DOI:** 10.1128/spectrum.00126-25

**Published:** 2025-06-12

**Authors:** Gourab Halder, Bhaskar Narayan Chaudhuri, Balaji Veeraraghavan, Priyanka Denny, Paulami Dutta, Mandira Chakraborty, Ujjwayini Ray Khan, Shelley Sharma Ganguly, Subhranshu Mandal, Yesha Parasmani Upadhyaya, Bedobroto Biswas, Arindam Chakraborty, Sourav Maiti, Himadri Mondal, Saikat Pal, Shanta Dutta

**Affiliations:** 1 Division of Bacteriology, ICMR-National Institute for Research in Bacterial Infections, Formerly ICMR-NICED, Kolkata, West Bengal, India; 2 Division of Microbiology, Peerless Hospitex Hospital, Kolkata, West Bengal, India; 3 Department of Clinical Microbiology, Christian Medical College 196380, Vellore, Tamil Nadu, India; 4 Collaborative Research Centre of Okayama University for Infectious Diseases in India at ICMR-NIRBI, Kolkata, West Bengal, India; 5 Department of Microbiology, Calcutta Medical College 30145, Kolkata, West Bengal, India; 6 Department of Microbiology, Apollo Gleneagles Hospital, Kolkata, West Bengal, India; 7 Department of Microbiology, Manipal Hospital, Formerly AMRI Hospital Saltake, Kolkata, West Bengal, India; 8 Department of Microbiology, CNCI 30153 , Kolkata, West Bengal, India; 9 Division of Microbiology, Desun Hospital 707509 , Kolkata, West Bengal, India; 10 Division of Microbiology, Fortis Hospital 422615, Anandapur, West Bengal, India; 11 Division of Microbiology, Ruby General Hospital 76253 , Kolkata, West Bengal, India; 12 Division of Microbiology, Manipal Hospital, Formerly Columbia Asia Hospital, Kolkata, West Bengal, India; 13 Department of Microbiology, Calcutta Medical Research Institute 78663 , Kolkata, West Bengal, India; Central Texas Veterans Health Care System, Temple, Texas, USA

**Keywords:** triple carbapenemases, *bla*_KPC-2_, ceftazidime-avibactam resistant *bla*_KPC-2_, *bla*_KPC-2 _with *bla*_NDM _, bla_KPC-2 _with bla_OXA-48-like-variants_, sepsis, ICU, MLST, novel ST, WGS, phylogenetic lineage, India

## Abstract

In view of insufficient data on the epidemiology and genomics of *bla*_KPC-2_ producing *Klebsiella pneumoniae* subsp. *pneumoniae* (KPC-2 *Kpn*) from India, this study, first of its kind, was conducted to characterize blood-borne KPC-2 *Kpn* isolates according to their resistome, plasmid types, and genotypes. This study included 45 blood-borne KPC-2 *Kpn* isolates from ICU-admitted sepsis patients in Kolkata from 2015 to 2024. These isolates were tested for antibiotic susceptibility against ≥10 antimicrobial classes. Antimicrobial resistance gene profiles, genetic environment of *bla*_KPC-2_, plasmid types, MLST genotypes, and virulence genes were determined by whole-genome shotgun sequencing (WGS). The transmissibility of *bla*_KPC-2_ genes was evaluated by conjugation experiment. Pulsed-field gel electrophoresis (PFGE) was conducted to confirm their clonality. SNP-based phylogenomic analysis was performed to compare the study isolates with global KPC-2 *Kpn* isolates. All 45 (100%) KPC-2 *Kpn* isolates showed resistance to 21 antimicrobials. In addition, 55.55% and 33.33% isolates were resistant to colistin and ceftazidime-avibactam, respectively. Presence of triple carbapenemase (*bla*_KPC-2_, *bla*_NDM-1/5_ and *bla*_OXA-181/232_) was observed in six isolates. *bla*_KPC-2_ transferable by IncFII/IncFII(pBK30683)/ColRNAI conjugative plasmids. WGS identified two distinct genetic environments of *bla*_KPC-2_ among the isolates based on presence or absence of Tn*4401*. PFGE identified 45 pulsotypes with a 39.3% similarity coefficient, indicating a variety of infection sources. Of the 16 MLST genotypes found in circulation, ST15 was predominant and ST7485 was novel. Hypervirulent genetic markers (*iucA*, *iutA*, and *rmpA2*) were identified in four isolates. Hospital infection control and surveillance programs should be strengthened to detect and combat *K. pneumoniae* co-harboring *bla*_KPC-2_ and/or *bla*_NDM_ and/or *bla*_OXA-48-like_ carbapenemase.

**IMPORTANCE:**

*Klebsiella pneumoniae* (*Kpn*) is the primary etiology of ICU-related bloodstream infections in India. Multi-drug resistance, including resistance to carbapenems and colistin, along with myriad virulence factors, makes this bacterium really troublesome with respect to effective treatment. In India, *bla*_NDM_ and *bla*_OXA-48-like_ carbapenemases are the most prevalent types with only sporadic reports of *bla*_KPC_ in *Kpn*. This study focuses on the emergence of *bla*_KPC-2_ in *Kpn* along with other carbapenemases in Kolkata, India. This study is also the first of its kind to describe the presence of triple carbapenemase (*bla*_KPC-2_, *bla*_NDM-1/5_ and *bla*_OXA-181/232_) and concurrent resistance to carbapenems, colistin, and ceftazidime-avibactam antimicrobials in *Kpn.* In addition, this study also highlights the diverse genotypes (sequence types) identified in KPC-2 *Kpn,* including the novel ST7485 identified in this study. Routine monitoring of antimicrobial resistance genes in *Kpn* should be encouraged to identify the emerging resistome, which will aid in determining the most effective treatment.

## INTRODUCTION

The global burden of illness and death caused by bloodstream infections (BSIs) is substantially high in both adults and children (1). Inadequate infection control measures, frequent use of invasive medical devices, severe infection, compromised host defenses, and prolonged exposure to antimicrobials contribute to increased risk of BSIs in patients admitted to the intensive care unit (ICU) (2). Approximately 5–15% of patients admitted to the ICU manifest BSIs during their initial month of admission, with case fatality ranging between 15% and 50% (3). In addition, BSIs caused by multi-drug-resistant bacteria impose a profound financial burden on the patient family as well as government due to increased costs, complications, length of stay in the ICU, and mortality rates (4–6).

The environmental or opportunistic pathogen *Klebsiella pneumoniae (K. pneumoniae*) is reported as the leading cause of BSIs caused by Gram-negative bacteria globally (1). In both immunocompromised and immune-sufficient persons, *K. pneumoniae* can cause a variety of illnesses, including pneumonia, UTIs, septicemia, and liver abscesses (7). In 2016, *K. pneumoniae* was identified as the third most common bacterium responsible for ICU-BSIs, according to the European Centre for Disease Prevention and Control (8). Between 1997 and 2016, the SENTRY Antimicrobial Surveillance Program noticed a steady rise in the prevalence of BSI caused by *K. pneumoniae* (9). As per the surveillance data from India, *K. pneumoniae* was documented as the second most prevalent GNB in the ICUs as well as the leading pathogen causing BSIs (10).

Multiple surveillance studies conducted during the 20th century across the globe have reported rising multi-drug resistance in *K. pneumoniae*, with 20–80% resistance toward first-line antibiotics, aminoglycosides, fluoroquinolones, and third-generation cephalosporins (11). The current treatment regimen for BSIs caused by *K. pneumoniae* includes the carbapenems. However, high rates of carbapenem resistance due to enzymes like New Delhi metallo-β-lactamase (NDM), VIM, *K. pneumoniae* carbapenemase (KPC), and OXA-48-like carbapenemases, along with increased virulence traits, have made treatment quite challenging for the health-care providers.

As per the resistance map of the Center for Disease Dynamics, Economics and Policy for India, carbapenem resistance in *K. pneumoniae* was recorded to be 59% in 2020. India, which is plagued by NDM and OXA-48-like carbapenemases, rarely reported KPC as the predominant carbapenemase in *K. pneumoniae*, which is in contrast to those documented in the United States, China, South America, and Europe (7). The enzyme KPC, as the name suggests, was first detected in *K. pneumoniae* isolates from the United States (North Carolina) in 2000, and since then, it has spread across the globe (12). Till date, 242 alleles of KPC have been reported (http://www.bldb.eu). KPCs are important with respect to public health due to their outstanding dissemination potential and the fact that they are frequently missed by standard susceptibility testing. Not only have these organisms created new problems with infection management, but there are also very few antibiotics available, making it extremely difficult for clinicians to treat illnesses caused by them (13). Infections caused by KPCs frequently result in fatalities ranging from 50% to 66% (7).

In India, studies centering around the genomic characteristics and epidemiology of *K. pneumoniae* isolates harboring *bla*_KPC_ genes in particular are scanty. This study, first of its kind, demonstrates the presence of *bla*_KPC-2_ producing *Klebsiella pneumoniae* subsp. *pneumoniae* (KPC-2 *Kpn*) in ICU units over a period of 10 years (2015–2024). The isolates were characterized in terms of their antimicrobial resistance (AMR) profiles, sequence types (STs), transmissibility of KPC, and associated mobile genetic elements. We also performed whole-genome shotgun sequencing (WGS)-based core genome analysis by including *K. pneumoniae* from another sporadic Indian study (7) as well as global isolates, to introspect their diverse carbapenemase lineages and global epidemiology.

## MATERIALS AND METHODS

### Clinical study isolates, phenotypic, and genotypic determination of ***KPC-2*** gene and their antibiogram

**Fig 1:**
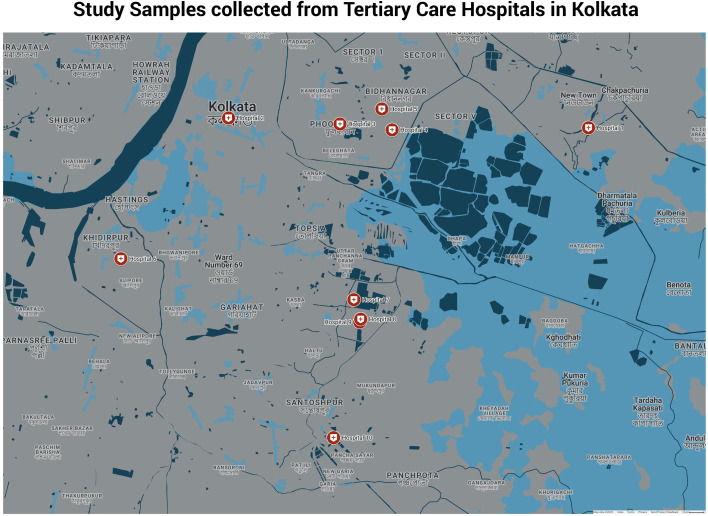
Geographical map of participating hospitals in Kolkata.

Carbapenem-resistant *K. pneumoniae* (CR-*Kpn*) prospectively collected from central-line blood of sepsis patients admitted in ICU of 10 different tertiary care hospitals in Kolkata were included in this study. The geographical map of the participating hospitals has been depicted in Fig. 1. Any patient who had received any form of carbapenem antibiotic prior to hospital admission was excluded from the study. The blood culture positive isolates were identified via MALDI-TOF-MS (bioMérieux, France) or VITEK-2 Compact (bioMérieux, France). Routine antimicrobial susceptibility tests by Kirby Bauer disc diffusion or VITEK-2 Compact were performed in the microbiology departments of respective hospitals. The CHROMagar or MacConkey agar plates with CR-*Kpn* were sent to ICMR-NIRBI for further investigation.

At ICMR-NIRBI, the isolates were reconfirmed by VITEK-2 Compact. The presence of the *bla*_KPC_ gene in the study isolates was phenotypically assayed by inhibitor-based boronic acid test following the protocol as given in an earlier study (14). The presence of the *bla*_KPC-2_ gene in the study isolates was confirmed by PCR (15, 16) and Sanger sequencing. The subsequent investigations were carried out only for isolates producing KPC-2. No other KPCs were detected.

Kirby Bauer disc diffusion and broth microdilution(BMD) assays for KPC-2 *Kpn* were done (17) to determine the antimicrobial susceptibility and MIC of resistant antimicrobials respectively in accordance with guidelines laid down by the Clinical and Laboratory Standards Institute, 2023 (18).

### WGS, core genome analysis, and generation of phylogenetic tree

Genomic DNA was isolated by Qiagen DNeasy Blood and Tissue Kit (Qiagen) and measured using Qubit 4.0 (Thermo Fisher Scientific, USA). The Nextera XT Kit aided in DNA library preparation; paired-end sequencing with a read length of 2 × 250 bp (base pairs) and a sequencing cycle of 2 × 501 was performed on the Illumina NovaSeq 6000 platform following sequencing protocol v1.5 chemistry provided by the manufacturer (Illumina Inc, San Diego, CA, USA). In addition, long-read sequences using SQK-LSK114 Ligation Sequencing Kit were also generated through Nanopore MinION (Oxford, UK) to comprehend the genetic environment of the *KPC* carbapenemase genes.

Both Unicycler (github.com/rrwick/Unicycler) and CLC Workbench Premium 22.0 (Qiagen) software enabled *de novo* assembly of long- and short-raw reads. The quality of the assembled contigs was assessed by QUAST v5.0.2 (http://quast.sourceforge.net/). All erroneous mappings in the contigs produced during the assembly were rectified by PILON v1.23 (http://github.com/broadinstitute/pilon/releases/). The RAST server, accessible at (http://rast.nmpdr.org) and Prokka (1.13) (https://github.com/tseemann/prokka), were used to facilitate the annotation of the genomes. The resulting .gbk file was visualized using Artemis.

The genome characteristics were determined by DFAST (https://dfast.ddbj.nig.ac.jp/). pubMLST (https://pubmlst.org/species-id) and ANI calculator enabled speciation of CR-*Kpn* (19). The online tool Resfinder version 4.7.2 (http://genepi.food.dtu.dk/resfinder) and PlasmidFinder version 2.1 (https://cge.food.dtu.dk/services/PlasmidFinder/) revealed the AMR genes and plasmid incompatibility (Inc) types respectively. The Institute Pasteur’s whole-genome MLST database (https://bigsdb.pasteur.fr/klebsiella/) along with the Kleborate pipeline (https://github.com/katholt/Kleborate) was used to assess both STs and virulence genes in the study isolates. The phylogenetic relatedness between the STs was determined by goeBURST and phyloviz software. The core genome phylogenetic tree comprising the study isolates and KPC-2 containing global isolates was built using IQ Tree (https://github.com/iqtree/iqtree2). For this, *K. pneumoniae* genomes (*n* = 48,000) were downloaded from NCBI (last accessed on 1 December 2024). The downloaded genomes were screened for the *bla*_KPC-2_ gene by Abricate (https://github.com/tseemann/abricate), using resfinder database (doi: https://doi.org/10.1093/jac/dks261) and thereafter, the STs of the KPC-2 positive global strains were determined by *in silico* MLST (https://github.com/tseemann/mlst). The presence of *bla*_KPC-2_ carbapenemase and the different STs as found in the study isolates were used as selection criteria for generating the tree. Information on the country and source of the isolate was retrieved from the BioSample database within NCBI. Additionally, raw sequencing reads were obtained from the ENA repository after a literature search, and a .fasta file was generated from it. The quality check, assembly, and annotation of all the downloaded .fasta files were done as mentioned earlier. The .gff files obtained (study isolates and selected global isolates) from Prokka were fed into Roary (v3.12.0) (https://github.com/sanger-pathogens/Roary) to generate an alignment of the core genome. The phylogenetic tree (.nwk file) was generated by IQ Tree (https://github.com/Cibiv/IQ-TREE) and visualized in Interactive Tree Of Life (https://itol.embl.de/). In addition, a heatmap was created encompassing carbapenem genes including *bla*_KPC-2_, STs, and other associated AMR genes in both study and global isolates.

### Plasmid isolation and conjugation assays

Plasmid isolation (≤50 kb) was done using QIAGEN Plasmid *Plus* Midi Kit (Qiagen, Germany) following the manufacturer’s instructions. The isolation of plasmids > 50 kb was done by the Kado and Liu method (20). The conjugal transferability of *bla*_KPC-2_ genes was assayed by liquid mating method as reported earlier (21) using wild study strains as donor and plasmid-free, sodium azide-resistant *Escherichia coli* J53 strain as recipient. The transconjugants (TCs) were selected on chrome agar plates containing 100 mg/L of sodium azide and varying concentrations of meropenem (ranging from 1 to 3 mg/L) (Sigma-Aldrich, St. Louis, MO, USA). The TCs were confirmed by biochemical profiling and VITEK 2. The MIC of carbapenems in TC was determined by BMD, while the transferred AMR genes were determined by PCR (21–25), followed by Sanger sequencing employing plasmid DNA of the TC as a template. Plasmid incompatibility types of the transferred plasmids were determined by PCR (26).

### Pulsed-field gel electrophoresis (PFGE)

The clonality among the KPC-2 *Kpn* isolates was deciphered by PFGE following standard protocol (21, 27). Bacterial DNA was digested with *Xba*I restriction digestion enzyme (50 U/plug), and PFGE was run for 21 h at 6 V/cm potential with switch times of 6 and 36 s in the CHEF-DRIII electrophoresis system (Bio-Rad Life Science Group, Hercules, CA, USA); using *K. pneumoniae* ATCC BAA 1705 as positive control and *Xba*I-digested *Salmonella* Braenderup H9812 as molecular weight ladder. FP-Quest Software, v4.5 (Bio-Rad Laboratories, Hercules, CA, USA) was used to construct the dendrogram based on the Dice coefficient with a band position tolerance of 1.5%. The band patterns were analysed in accordance with earlier suggestions (28).

## RESULTS

### CR-*Kpn*

**Fig 2:**
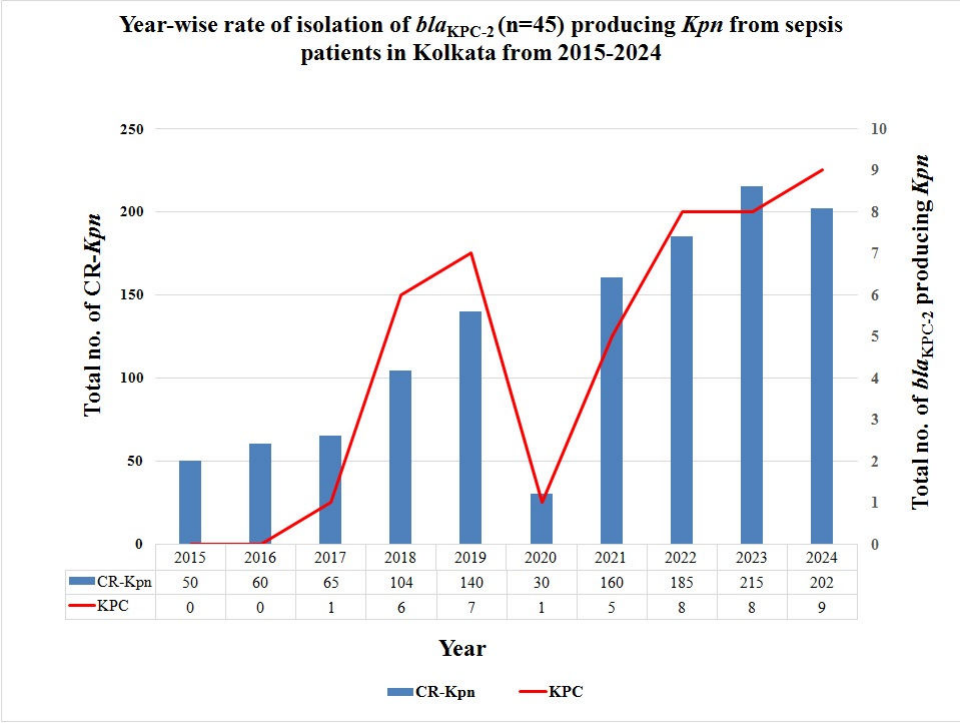
The year-wise rate of isolation of *bla*_KPC-2_ (*n* = 45) producing *Kpn* from sepsis patients admitted in 10 different tertiary care hospitals in Kolkata from 2015 to 2024.

Between January 2015 and December 2024, a total of *n* = 1,211 non-duplicative CR-*Kpn* blood isolates were collected from 10 participant hospitals in Kolkata and included in this study. *bla*_KPC-2_ were found in *n* = 45 (45/1,211 = 3.71%) isolates by PCR. In this study, the *bla*_KPC-2_ harboring CR-*Kpn* isolate was first found to emerge in 2017. Thereafter, it was isolated every year; however, the rate of isolation differed with each year, as shown in Fig. 2. These 45 isolates were found to co-harbor other carbapenemases in several combinations by WGS. Among these, double carbapenemases were found in 16 isolates (*bla*_KPC-2_ and *bla*_OXA-232_ were found in *n* = 7 [7/45 = 15.55%]; *bla*_KPC-2_ and *bla*_NDM-5_ in *n* = 5 [5/45 = 11.11%]; *bla*_KPC-2_ and *bla*_NDM-1_ in *n* = 4 [4/45 = 8.88%]), while 6 isolates produced triple carbapenemases (*bla*_KPC-2_, *bla*_NDM-1_ and *bla*_OXA-181_ in *n* = 3 [3/45 = 6.66%], *bla*_KPC-2_, *bla*_NDM-5_ and *bla*_OXA-232_ in *n* = 2 [4.44%], *bla*_KPC-2_, *bla*_NDM-5_ and *bla*_OXA-181_ in *n* = 1 [1/45 = 2.22%]). Carbapenemases like *bla*_IMP_ and *bla*_SIM_ were not found in them.

### AMR in KPC-2 *Kpn*

All 45 (100%) KPC-2-*Kpn* isolates were resistant to 10 antimicrobial classes encompassing 21 antimicrobials namely doripenem, imipenem, meropenem, and ertapenem (carbapenems); ceftaroline, cefepime, cefotaxime, and ceftazidime (cephalosporins); aztreonam (monobactam), piperacillin-tazobactam, amoxycillin-clavulinic acid, and ampicillin-Sulbactam (β-lactam combination); ciprofloxacin, levofloxacin, ofloxacin (fluoroquinolones), Fosfomycin, and gentamicin (aminoglycoside); and trimethoprim-sulfamethoxazole (folate pathway blockers), chloramphenicol (phenicol), tetracycline, and ampicillin (penicillin). In addition, colistin resistance was observed in *n* = 25 (55.55%) isolates. Resistance to ceftazidime-avibactam (Caz-Avi) was observed in *n* = 15 (33.33%) isolates. Interestingly, these 15 study isolates co-harbored *bla*_NDM_ (*bla*_NDM-1_ or *bla*_NDM-5_) and *bla*_KPC-2_. The isolates (*n* = 30) which produced *bla*_KPC-2_ alone or in combination with *bla*_OXA-48 like variant_ were found to be susceptible to Caz-Avi. Tigecycline and minocycline were the two antibiotics to which all 45 study isolates were uniformly susceptible. The percentage resistance, as well as MIC_50_ and MIC_90_, for different antibiotics are shown in Table 1.

**TABLE 1:** MIC ranges, MIC_50_, and MIC_90_ of different antimicrobials in *bla*_KPC-2_-producing *Klebsiella pneumoniae* isolates (*n* = 45)

Antimicrobial agents	Minimum inhibitory concentration (MIC) (mg/L)			
	Range of MIC	MIC_50_	MIC_90_	% of Resistance
Carbapenems				
Imipenem	16–≥256	16	32	100
Meropenem	32–≥256	32	64	100
Ertapenem	8–≥256	32	16	100
Doripenem	16–512	64	16	100
Cephems (Cephalosporins)				
Ceftazidime	512–≥1,024	1,024	512	100
Cefotaxime	256–≥1,024	512	512	100
Cefepime	64–512	128	64	100
Ceftaroline	32–128	128	32	100
Monobactams				
Aztreonam	64–≥512	512	256	100
β-Lactam combination agents				
Ampicillin-Sulbactam	128/64–256/32	256/32	256/32	100
Amoxicillin-Clavulanic acid	≥128/64–512/32	256/64	128/32	100
Piperacillin-Tazobactam	≥32/4–512/4	256/32	64/8	100
Ceftazidime-Avibactam^*b*^	32/4–128/32	ND^*a*^	ND^*a*^	15 (33.33)
Aminoglycosides				
Gentamicin	32–128	128	64	100
Fluoroquinolones				
Ciprofloxacin	8–256	16	8	100
Levofloxacin	4–512	8	4	100
Ofloxacin	8–32	8	8	100
Folate pathway antagonists				
Trimethoprim-Sulfamethoxazole	8/152–256/4,864	64/302	16/228	100
Phenicol				
Chloramphenicol	64–512	256	64	100
Tetracyclines				
Tetracycline	32–256	128	64	100
Lipopeptides				
Colistin^*b*^	4–64	ND^*a*^	ND^*a*^	25 (55.55)
Fosfomycins				
Fosfomycin	256–512	256	256	100

^*a*^ ND, not done.

^*b*^ Since a lower number of isolates with high MIC could affect the final calculation, the MIC_50_ and MIC_90_ for ceftazidim-avibactam and colistin were not carried out.

### Resistome analysis of KPC-2 *Kpn*

**Fig 3:**
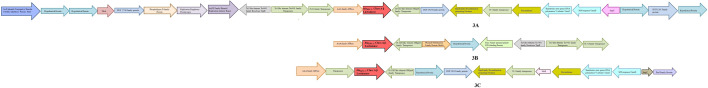
Genetic environment of *bla*_KPC-2_ gene in KPC-*Kpn* study isolates. Presence (A) and absence of Tn*4401* (B and **C**) upstream of *bla*_KPC-2_ gene in KPC-*Kpn* study isolates.

WGS analysis showed the presence of only one type of *bla*_KPC_, namely *bla*_KPC-2_, in all 45 KPC-*Kpn* isolates. Analyses of the genetic environment of the *bla*_KPC-2_ genome revealed two major structural patterns based on presence or absence of the Tn*4401* transposase element (Fig. 3). In the majority (*n* = 40) of the isolates, the Tn*4401* was present upstream of *bla*_KPC-2_ (Tn*4401*-IS*21* family transposase-AAA family ATPase-*bla*_KPC-2_-IS*1182* family transposase) (Fig. 3A). In others where Tn*4401* was absent, two variations were discovered as follows: AAAfamily ATPase-*bla*_KPC-2_-IS*Kpn6* family transposase (Fig. 3B) and AAAfamily ATPase- Transposase-*bla*_KPC-2_-IS*Kpn6* family transposase (Fig. 3C).

**Fig 4:**
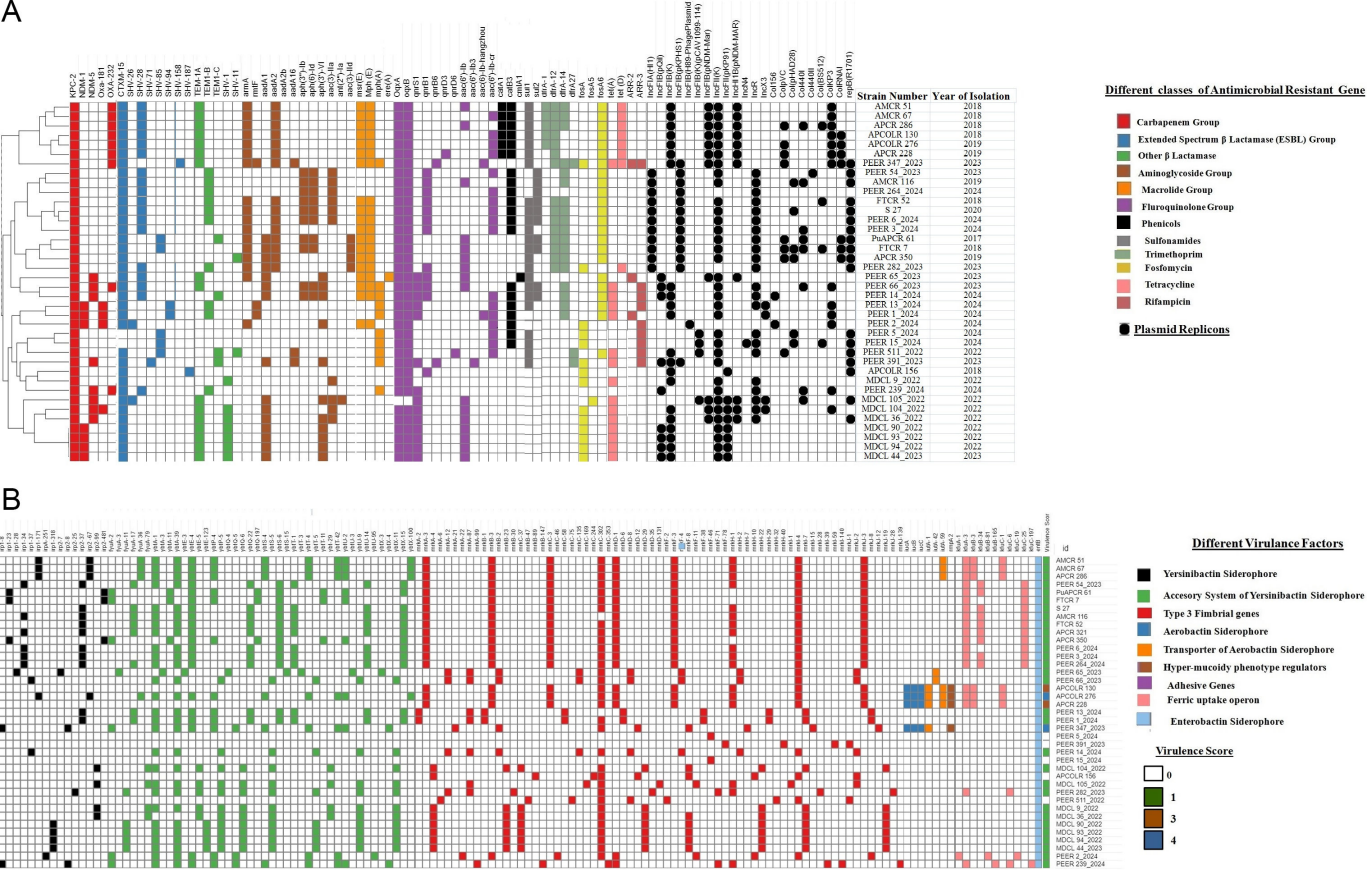
(**A**) Heatmap of KPC-2 *Kpn* study isolates depicting respective AMR genes and plasmid replicon typing. Cluster analysis was performed on the heatmap using Euclidean distance. The filled boxes and circles represent the presence, and no fill represents the absence. (**B**) Heatmap depicting the presence or absence of different virulence determinants found in KPC-2 *Kpn* study isolates.

Further, the resistome of KPC-2 *Kpn* isolates also comprised several ESBL genes like *bla*_SHV_ (variety of alleles), *bla*_OXA-1_, and *bla*_OXA-9_ with or without *bla*_CTX-M-15_. In addition, several other β-lactamases, aminoglycosides, fluoroquinolones, and other AMR genes, as illustrated in the heatmap (Fig. 4A), were also present. Fluoroquinolone resistance in 15 study isolates was attributed to double mutations in the *gyrA* gene (S83F and D87A [*n* = 9]; S83I and D87G [*n* = 3]; S83Y and D87G [*n* = 3]) along with a single *parC* (S80I) mutation. In remaining isolates, a single mutation both in *gyrA* (S83Y) and *par*C (S80I) was found.

Although 25 study isolates were colistin resistant, none possessed the plasmid-mediated *mcr* genes. However, a variety of mutations were found in all the isolates in *phoQ* (R16L), *phoP* (S71L), *pmrA* (E57G), *pmrC* (S257L and F27C), *araN* (Q211L and G301R) either alone or in different combinations. In addition, complete deletion of the *mgrB* gene along with double mutation (F27C and S257L) in *pmrC* and single mutation (Q211L) in *araN* were observed in two isolates. Further, four colistin-resistant isolates showed deletion of seven amino acids MKKLRWV in *mgrB* along with single mutations F27C and Q211L in *pmrC* and *araN*, respectively.

Among the many mechanisms responsible for Caz-Avi resistance in KPC-*Kpn*, sequence analysis of 15 caz-avi resistant study isolates revealed multiple mutations in the *ftsI* gene and complete deletion of OmpK36 porin in all isolates. Additionally, four isolates showed an 82 amino acid deletion in OMPK35 porin, while one isolate showed L192R substitution in the *KPC-2* gene.

### Virulome analysis of KPC-2 *Kpn*

A multitude of virulence factors including *kfu* gene (iron-acquisition system). *irp-1* gene (yersinibactin siderophore), *ybt* and *fyu* genes (yersinibactin transporter), *mrkABCD* gene cluster (type 3 fimbriae), *iucABCD* gene (aerobactin), *iutA* (transporter of *iucABCD* gene cluster), *rmpA* and *rmpA2* genes (regulator of capsular polysaccharide synthesis) were found in the study isolates and are depicted in the heatmap (Fig. 4B).

### Plasmid analysis of KPC-2 *Kpn*

**Fig 5:**
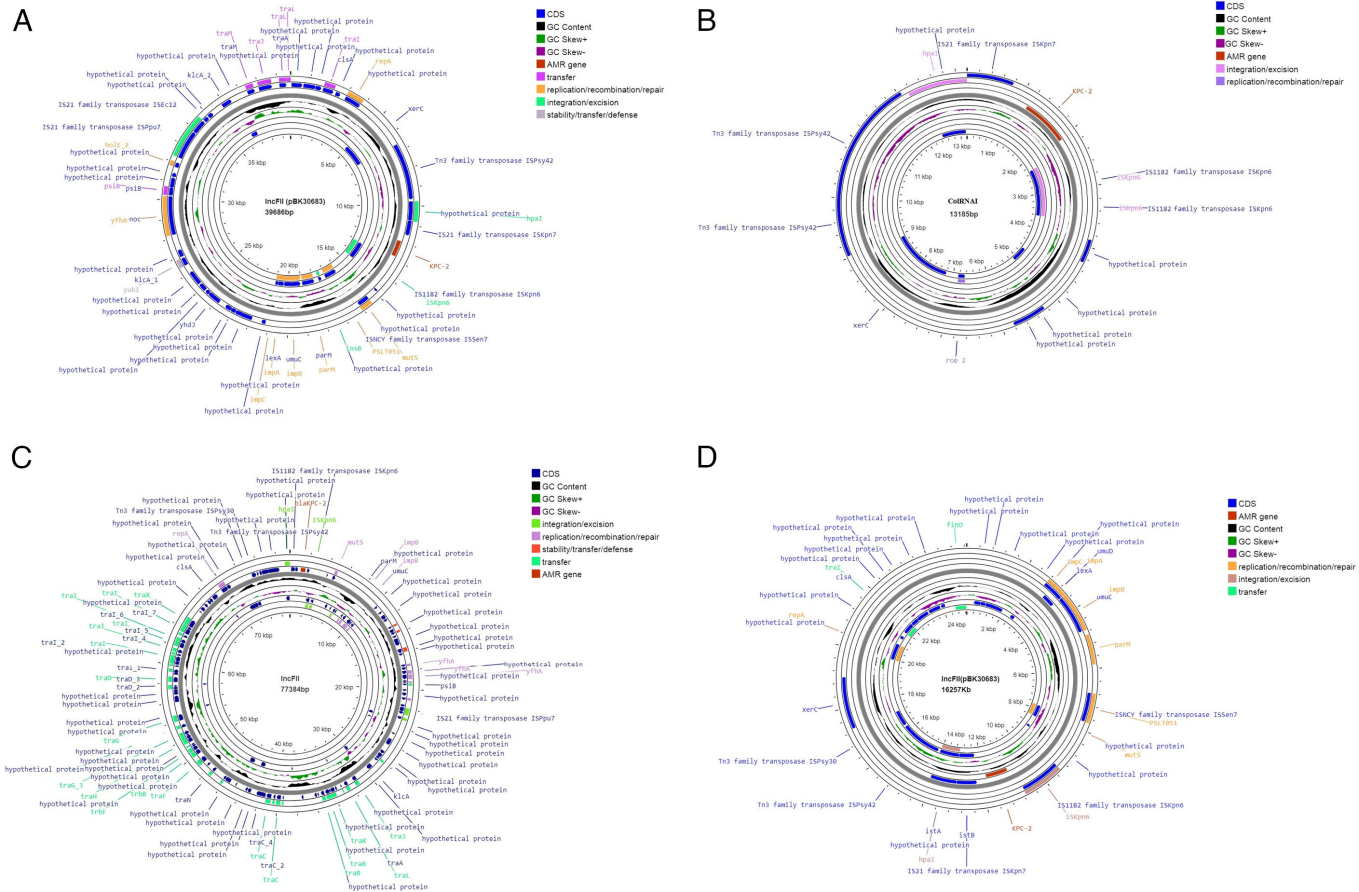
Circular map of different transmissible plasmids harboring *bla*_KPC-2_ carbapenemase genes. (**A**) IncFII (pBK30683) transmissible plasmid (39 kb) harboring *bla_KPC-2_* in KPC-2 *Kpn* study isolate (ST14). (**B**) ColRNAI replicon type transmissible plasmid (13 kb) co-harboring *bla*_KPC-2_ (ST2096). (**C**) IncFII transmissible plasmid (77 kb) harboring *bla_KPC-2_* in KPC-2 *Kpn* study isolate (ST15). (**D**) IncFII (pBK30683) transmissible plasmid (16 kb) harboring *bla*_KPC-2_ in KPC-2 *Kpn* study isolate (ST15).

The plasmid profiles of the KPC-2 *Kpn* study isolates were highly diverse, and each isolate had at least one plasmid ranging from 15 to 80 kb in size. Among the 22 plasmid types found in circulation by WGS (Fig. 4A), IncFIIK (*n* = 35) was found to be most prevalent. No correlation was found between the types of plasmids and specific AMR gene profiles. Also, none of the isolates harbored two carbapenemases on the same plasmid. The *bla*_KPC-2_ gene in the study isolates was found to be transferable via plasmids (size ≥13 kb) of three different incompatibility types IncFII or IncFII(pBK30683) or ColRNAI (Fig. 5A through D). Interestingly, in all six isolates with triple carbapenemases, the *bla*_KPC-2_ gene was transferred via IncFII (pBK30683) plasmid, *bla*_NDM_ via IncF(II)K plasmid, and *bla*_OXA-48-like_ variant via IncX3/ColKP3 plasmids.

### Multi-locus sequence typing (MLST)

**Fig 6:**
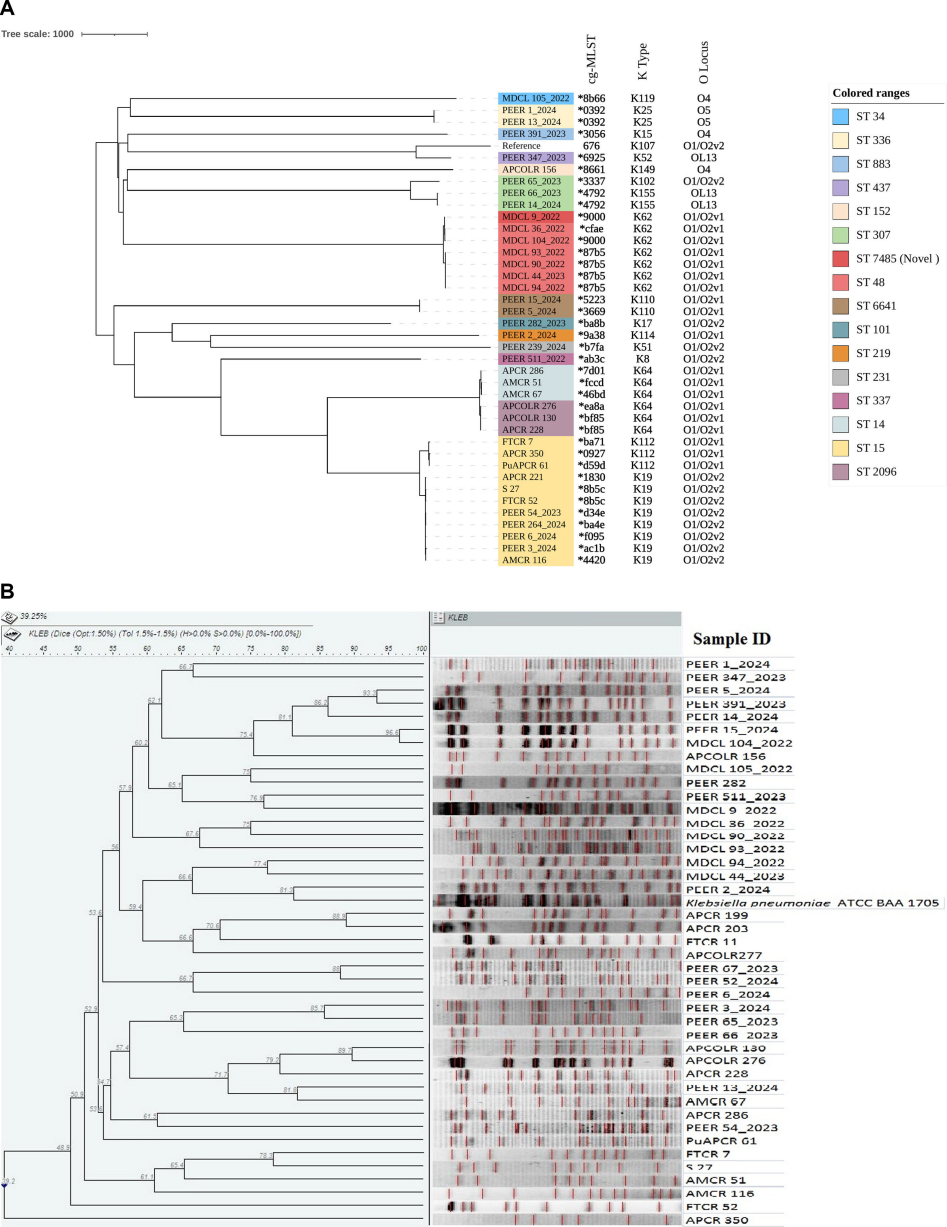
Comparative dendrograms of KPC-2 *Kpn* study isolates based on (**A**) WGS-based core genome SNP analysis and (**B**) PFGE.

In accordance with the MLST database, the KPC-*Kpn* study isolates were grouped into 16 separate STs, namely ST15 (*n* = 15); ST48 (*n* = 6), ST14 (*n* = 3); ST2096 (*n* = 3); ST307 (*n* = 4); ST6641 (*n* = 3); ST336 (*n* = 2); and ST34, ST101, ST152, ST219, ST231, ST337, ST437, ST883, and ST7485 (*n* = 1 each) (Fig. 6A). Among these, the ST7485 was a novel ST and was found to be closely related to ST48 as per goeBURST’s analysis of the molecular epidemiological linkages between the STs because it differed from ST48 by only one locus (*rpoB*). While ST336 (*n* = 2), ST219 (*n* = 1), and ST231 (*n* = 1) were exclusively associated with isolates producing triple carbapenemases, ST48 and ST307 genotype were shown by isolates producing both triple and dual carbapenemases in this study. Other STs also associated with dual carbapenemase in this study were ST14, ST437, ST34, and ST883.

### PFGE

The presence of 45 pulsotypes in PFGE was discovered employing restriction digestion of 45 KPC-2 *Kpn* isolates with *Xba*I, which culminated in a similarity coefficient of 39.25% (Fig. 6B). Each isolate had a different pulsotype, and the isolates belonging to the same STs also did not group under any cluster. There was an absence of any correlation between the strain pulsotypes and the hospitals. Further, no single pulsotype was found to be more prevalent than any other in any of the hospitals. Phylogenetically divergent KPC-2 *Kpn* strains in circulation indicate a wide array of infection reservoirs and an increased likelihood of KPC-2 *Kpn* strain dissemination.

### Phylogenetic tree analysis

**Fig 7:**
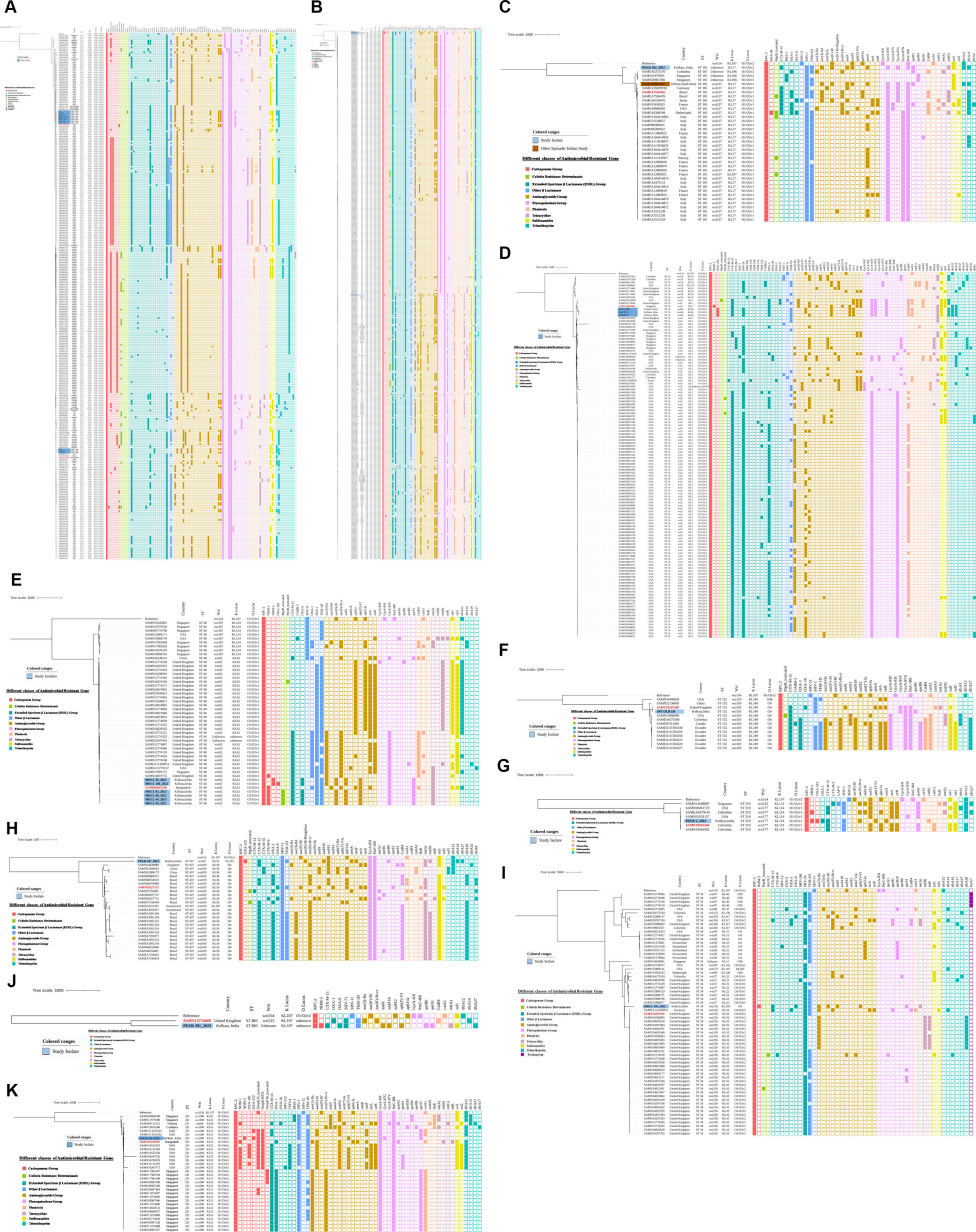
Pictorial depiction of phylogenomic SNP analysis of KPC-2 *Kpn* study isolates and their comparison with global isolates belonging to ST15 (**A**), ST307 (**B**), ST101 (**C**), ST14 (**D**), ST48 (**E**), ST152 (**F**), ST219 (**G**), ST437 (**H**), ST34 (**I**), ST883 (**J**), and ST231 (**K**). The dendrogram shows Country of origin, Wzi allele, K Locus, O Locus, and a heatmap indicating the presence or absence of KPC and other classes of antimicrobial genes. Study isolates are highlighted in light blue color and the global strain to which it is closely related is marked in deep red color.

According to the NCBI database, of the 16 MLST genotypes identified in this study, only 11 STs (ST14, ST15, ST34, ST48, ST101, ST152, ST219, ST231, ST307, ST437, and ST883) were associated with the *bla*_KPC-2_ gene. The ST7485 was a novel genotype discovered in this study. The remaining four STs (ST336, ST337, ST2096, and ST6641), although listed in GenBank, were associated with the NDM genotype and/or other carbapenemases (absence of KPC-2 carbapenemase). Hence, phylogenetic trees encompassing the aforesaid 11 STs were constructed (Fig. 7A through K) to understand the genetic lineage of Indian KPC-2 *Kpn* study isolates in reference to global isolates. Although all the STs showed diversity in their phylogeny, isolates belonging to ST34 were found to be more diverse due to 4,444 SNPs difference among them in comparison to SNPs difference among other STs: ST883 (2,856 SNPs), ST14 (1,478 SNPs), ST219 (1,313 SNPs), ST152 (1,100 SNPs), ST231 (1,079 SNPs), ST307 (1,005 SNPs), ST101 (945 SNPs), ST48 (789 SNPs), ST15 (646 SNPs), and ST437 (601 SNPs).

## DISCUSSION

The mechanism of resistance and molecular subtypes of the *Kpn* blood isolates that produce *bla*_KPC-2_ along with other carbapenemase genes that are emerging in India are thoroughly described in this study. In this study, KPC-2 *Kpn* was first isolated in 2017. In the past, a case report of *Kpn* co-harboring *bla*_KPC-2_ and *bla*_NDM-1_ isolated from Chennai, India, was reported in 2010 (28). A sporadic case of KPC-2 *Kpn* was also reported in 2017 from South India (7). It was interesting to note that all reports of KPC-2 Kpn from *Kpn* from India were from 2010 onwards. On the contrary, endemic and sporadic reports of KPC-2 *Kpn* have been documented from many countries like the United States, Canada, Italy, Argentina, Brazil, France, Greece, Colombia, Israel, and China since the 2000s (29, 30). In recent years, *K. pneumoniae* co-harboring *bla*_KPC-2_ and other carbapenemases has become quite common and has been documented across the globe including Italy (KPC-2/VIM-1) (29, 31), Colombia (KPC-2/VIM-24) (32), China (KPC-2/NDM-1, KPC-2/IMP-26 and KPC-2/IMP-4) (33–35), Pakistan (KPC-2/NDM-1) (36), and Greece (KPC-2/VIM-1) (37). We report the co-occurrence of *bla*_KPC-2_ along with *bla*_OXA-181_ and/or *bla*_OXA232_ and/or *bla*_NDM-5_ in *Kpn,* which is probably the first of its kind from India. Furthermore, the co-existence of three major carbapenemases (*bla*_KPC-2_, *bla*_NDM-1/5_ and *bla*_OXA-181/232_) in a single isolate, as found in this study, has never been documented from India so far. In this study, *Kpn* producing dual and triple carbapenemases were first isolated in 2018 and 2022, respectively (Fig. 4A)

The KPC-2 *Kpn* study isolates (*n* = 45) showed extreme antibiotic resistance to 21 antimicrobials. The MIC of the carbapenems tested ranged from (Table 1). No significant change in MIC of the carbapenems was observed in isolates producing single, dual, or triple carbapenemases (data not shown). In addition, resistance to colistin (MIC 4–64 mg/L) and Caz-Avi (MIC 32/4–128/32 mg/L) was exhibited by 55.55% and 33.3% of the isolates, which was not documented earlier from India and is extremely alarming from a public health point of view. Resistance to both these antimicrobials was first noted in 2018 in the study isolate AMCR51 harboring dual carbapenemase (Fig. 4A). Colistin resistance in *Enterobacteriaceae* has been linked to numerous mechanisms like the presence of plasmid-mediated *mcr* gene (38); chromosomal mutation and/or upregulation of two-component system (*phoP/Q* and *pmrA/B*) (39); complete deletion or truncation of *mgrB* (negative regulator of two-component system *PhoP/Q*); and upregulation of *arnABCD* operon (40). The colistin resistance in the KPC-*Kpn* study isolates could be attributed to the multiple mutations in *phoP/Q*, *pmrA/C,* and/or *arnA,* as well as complete deletion or truncation of *mgrB* found in them. Caz-Avi used in the treatment of KPC-*Kpn* is rendered ineffective by various mechanisms like truncation or mutation in porins like OmpK35, OmpK36, and LamB (41); mutations in *ftsI* (encoding PBP3 protein) (42); and mutations in the *bla*_KPC-2_ gene. Caz-Avi resistance in study isolates could be ascribed to multiple mutations observed in the *ftsI* gene, complete deletion of OmpK36 porin, 82 aa deletion in OmpK35, or absence of *LamB* porin.

The prominent genotypes interrelated with KPC-*Kpn* isolates worldwide include ST11, ST15, ST147, ST258, and ST307; of these, ST11, ST258, and ST307 were geographically confined to China (43), the United States (44), and South Korea (45–47), respectively. Among the aforesaid international clones, only ST15 and ST307 were found in this study. Phylogenetic analysis of eleven ST15 study isolates revealed that all shared close genetic relation with clinical strains from the United Arab Emirates and the United Kingdom (Fig. 7A). While eight study isolates (same cluster) were closely related (eight SNPs) to one unspecified human clinical sample of the United Arab Emirates (accession number SAMEA13607439), the other three study isolates (same cluster) differed by 52 SNPs from a strain isolated from human swab in 2017 in the United Kingdom (SAMEA11351073) (Fig. 7A). Similar to our study isolate, all global isolates in the phylogenetic tree showed the presence of *bla*_KPC-2_ only, except for two isolates (SAMN26422232 and SAMN26422228, differing by 60–216 SNPs and 58–214 SNPs, respectively, from our study strains) from Turkey, which had *bla*_KPC-2_ along with bla_NDM-1_ (Fig. 7A). Among the three ST307 study isolates, two (same cluster) were closely related (677 SNPs) to a clinical strain from the United Kingdom (SAMN12774184), while one shared homology (3 SNPs) with a strain from the Netherlands (SAMEA6368677) (Fig. 7B). In contrast to our study, the majority of ST307 global isolates were found to produce only *bla*_KPC-2_. Isolation of KPC-2 *Kpn* belonging to ST101 as found in this study, was also documented from South India earlier. So, a phylogenetic tree was constructed to determine their relatedness (Fig. 7C). But surprisingly, the study isolate was found to be closely related to a Brazilian strain (702 SNP difference) isolated from human blood in 2016 (SAMEA7556456), but more distantly related (724 SNP difference) to the strain from South India (SAMN06885836). In tandem with our study, all global isolates showed the presence of only the *bla*_KPC-2_ gene, except for one strain from the Netherlands. SNP analysis of other genotypes revealed that ST14 study isolates (Fig. 7D) were in close relation to a Singaporean clinical strain (SAMN14649904) with 171 SNPs difference and both being dual carbapenemase producers; ST48 study isolate (Fig. 7E) was closest (15 SNPs) to a non-clinical Bangladeshi strain (SAMD00607298) which also showed presence of dual carbapenemase like the study isolates. Interestingly, the majority of the global isolates have reported only *bla*_KPC-2_ in them. ST219 (Fig. 7G) study isolate shared homology (24 SNPs) with a Colombian clinical strain (SAMN19016344), but unlike the study isolate which produced triple carbapenemase, the Colombian strain possessed only *bla*_KPC-2_; ST437 study isolate (Figure 7H) with 548 SNP difference was more close to a Brazilian clinical isolate (SAMN02927713), but unlike the study isolate which produced dual carbapenemase, the Brazilian strain possessed only *bla*_KPC-2_. Further study isolates belonging to ST152 (Fig. 7F), ST34 (Fig. 7I), and ST883 (Fig. 7J) all were found to be phylogenetically close to clinical strains from the United Kingdom with SNP differences of 45 (SAMN24297269), 995 (SAMN24297010), and 2,856 (SAMN12774468), respectively. Interestingly, one study isolate harboring triple carbapenemase *bla*_KPC-2_, *bla*_NDM-5_, *bla*_OXA-232_ and belonging to ST 231 was found to be more closely related to a Bangladeshi strain (SAMN29188276 with 58 SNP differences) with dual carbapenemase *bla*_KPC-2_ with *bla*_NDM-1_ than the strain from the United States (SAMN30107552 with 891 SNP differences) producing triple carbapenemase *bla*_KPC-2_, *bla*_NDM-1_ and *bla*_OXA-181_ (Fig. 7K).

Phylogenetic analysis by WGS-based SNPs clustered the study isolates on the basis of their STs (Fig. 6A), which was absent in PFGE analysis (Fig. 6B), reconfirming that WGS-based SNP analysis is ideal for deciphering genetic correlation.

From a virulence point of view, hypervirulent *Kpn* (hv*Kpn*) harboring a large virulence plasmid (pLVPK-like) encoding several virulence genes including genes for regulation of capsular polysaccharide synthesis (*rmpA* and *rmpA2*) and siderophores like aerobactin for iron sequestration (*iucABC* and *iutA*) and salmochelin (*iroBCDN*) has been in circulation for a long time (48, 49). But the earlier strains were susceptible to common antimicrobials. In the current scenario, strains carrying both hypervirulent gene markers and carbapenem resistance are on the rise and pose a great threat with respect to treatment options (50, 51). The definition of hypervirulence is, however, debatable because while some researchers have suggested the presence of all these markers (*rmpA*, *rmpA2*, *iro*, *iuc*, and/or *peg-344*) (52), others consider *iucA* as the most accurate virulence marker for hypervirulent strain identification (50). In this study, genes like *iucABC*, *iutA*, and *rmpA2* were present in four isolates (Fig. 4B), indicating the circulation of hypervirulent strains in this region. Unfortunately, these four strains were positive for both *bla*_KPC-2_ and *bla*_OXA-232_ genes, similar to other reports (53, 54), which warrants immediate and strict surveillance to curb the dissemination of these notorious pathogens.

This study is not without limitations. Foremost, the study isolates were collected from the Kolkata region only and therefore undermine the true burden of KPC-*Kpn* among the Indian population. A collaborative study involving KPC-*Kpn* isolated from pan India hospitals would greatly help in understanding the epidemiology and emerging genotypic profiles. Furthermore, assessing the role of different mutations found in two-component system genes and porins related to colistin and Caz-Avi resistance in the study isolates was beyond the scope of this study. Moreover, only the STs found in this study were subjected to phylogenetic tree analysis, rather than all of the STs reported to harbor the *bla*_KPC-2_ worldwide.

### Conclusion

The emergence of *bla*_KPC-2_ along with other carbapenemases like *bla*_NDM_ and *bla*_OXA-48-like_ variants in *K. pneumoniae* is a serious health hazard in vulnerable populations, especially in ICU patients. The development of resistance to Caz-Avi and colistin in KPC-2 *Kpn* has profoundly restricted the alternatives for effective antimicrobial treatment, leading to an increase in clinical complications, morbidity, and mortality. The already existing threat of AMR in the community is exacerbated by the presence of easily transmissible plasmids that carry both resistance and virulence genes. Additionally, source tracking is made difficult by the phylogenetic diversity of KPC-2 *Kpn*. Therefore, in order to combat the spread of KPC-2 *Kpn*, it is imperative that hospital infection control and surveillance programs be enhanced.

## Data Availability

All genome sequences have been submitted NCBI database under Bioproject number PRJNA649689.

## AFTER EPUB

*[This article was published on 12 June 2025 with errors in the last affiliation, figure captions, and text. The affiliation, figure captions, and text were corrected in the current version, posted on 18 June 2025.]*
